# Supplementary figures and images for: Invasive Fungal Diseases in Hospitalized Patients with COVID-19 in Israel: A Multicenter Cohort Study
Source: J Fungi (Basel). 2022 Jul 9;8(7):721. doi: 10.3390/jof8070721 (PMC9317957; doi:10.3390/jof8070721)

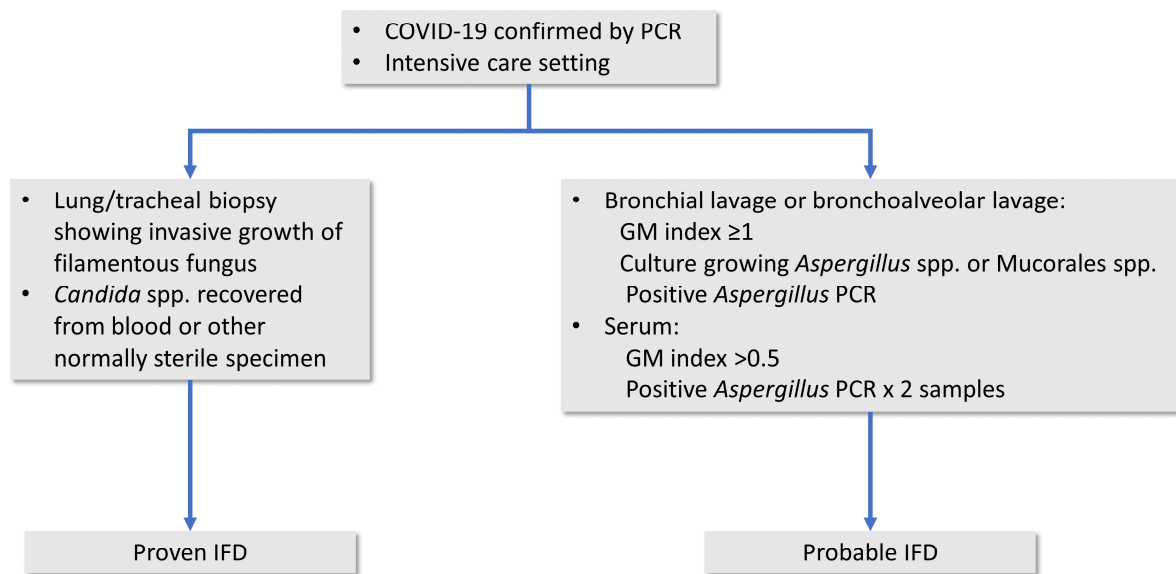

**Figure S1.** Workflow for defining invasive fungal diseases.

Supplement: Supplementary file 1 [file jof-08-00721-s001.zip › jof-1788950-SI.pdf]
